# Vaccine Liability in the Light of Covid-19: A Defence of Risk–Benefit

**DOI:** 10.1093/medlaw/fwab053

**Published:** 2022-01-13

**Authors:** Richard Goldberg

**Affiliations:** Durham Law School, Durham University, The Palatine Centre, Durham City DH1 3LE, UK

**Keywords:** COVID-19, medicinal products, product liability, risk–benefit, tort, vaccines

## Abstract

Vaccines have played an essential role in advancing medical treatment in the twentieth and twenty-first centuries. However, no medical intervention is risk free, and vaccines are no exception to that rule. This article considers how lawyers have confronted or eschewed risk–benefit in the context of determining defectiveness in vaccine liability, with emphasis on the UK, European Union, and US experiences. It explores the potential role that risk–benefit may play in assessing liability for vaccines against the COVID-19 pandemic. It argues that a holistic, flexible approach to determining defectiveness embracing risk–benefit allows consideration of the overwhelming public interest derived from the continued availability and supply of vaccines, as well as immunity conferring benefits on both the individual and the community. If cases do emerge concerning the liability of a COVID-19 vaccine, immunity conferring benefits on both the individual and the community of the COVID-19 vaccines should be relevant in any determination of defectiveness. Such a holistic, flexible approach to defectiveness embracing risk–benefit can be used effectively to determine the entitled safety of a vaccine and may help to mitigate against the dangers of weakening confidence in the public’s vaccine uptake.

## I. INTRODUCTION

Vaccines have played an essential role in advancing medical treatment in the 20th and 21st centuries. As Sir Jeremy Farrer has observed in the largest study to date into global attitudes to science and health, vaccines ‘are our most powerful public health tools’.^1^ However, no medical intervention is risk free, and vaccines are no exception to that rule.^2^

Section II addresses the issue of how the benefit–risk balance of vaccines is currently evaluated by regulatory agencies in the determination of a vaccine’s safety, and the continued need for improved communication of benefit–risk evaluation between decision makers and stakeholders. Section III then considers how lawyers have confronted or eschewed risk–benefit in the context of determining defectiveness in vaccine liability, with emphasis on the UK, European Union (EU), and US experiences. It then proceeds to examine the potential role that risk–benefit may play in assessing liability for vaccines against the Coronavirus disease (COVID-19) pandemic. It argues that a holistic, flexible approach to defectiveness embracing risk–benefit is the best way forward in this context, in that it allows consideration of the overwhelming public interest derived from the continued availability and supply of vaccines, as well as immunity conferring benefits on both the individual and the community.

In the light of the pandemic, Section IV concludes with a call for the overall risks and benefits of vaccines not to be marginalised by product liability lawyers. It cautions that the omission of risk–benefit from the court’s determination of defectiveness would be to ignore the iterative approach to benefit–risk analysis which is crucial to the determination of a vaccine’s safety, as well as potentially weakening confidence in the public’s vaccine uptake.

## II. BENEFIT–RISK EVALUATION OF VACCINES

### A. Benefit–Risk Assessment

As with all medicinal products, vaccines cannot be absolutely risk free. Every administration of a vaccine will carry the risk of an adverse event following it, which is not necessarily caused by the vaccine itself: *post hoc* is not *propter hoc*.^3^ While risk ratios or relative risks are helpful in assessing causality, decisions to approve vaccines are ultimately based on weighing the risks and benefits of the vaccine.^4^ An EU marketing authorisation for a vaccine shall be refused if the risk–benefit balance is not considered to be favourable.^5^ The risk–benefit balance is an evaluation of the positive therapeutic effects of the vaccine in relation to the risks concerning its quality, safety, or efficacy as regards patients’ health or public health.^6^ For every vaccine to be granted a UK marketing authorisation, it must be determined by the licensing authority that ‘the positive therapeutic effects of the product outweigh the risk to the health of patients or of the public associated with the product’.^7^ In the USA, all human drugs and biological products must be safe to be approved for marketing, and while ‘safe’ is not explicitly defined in statutes or regulations governing approval, the safety of a drug is determined by whether its benefits outweigh its risks.^8^ The UK and EU and US determinations all take the form of a benefit–risk assessment.

### B. Benefit–Risk Methodology

While the majority of regulatory decisions have traditionally been based on collective judgement,^9^ the last 20 years have seen a shift in approach to evaluating the benefit–risk profiles of medicinal products from a hitherto unstructured, subjective, and inconsistent approach to a more structured and objective process.^10^

The European Medicines Agency (EMA), aware of the importance of ensuring transparency and consistency across various stakeholders, commenced a 3-year Benefit–Risk Methodology project in 2009, which in its final form suggested a two-level approach to evaluations: (i) a qualitative approach with no quantitative modelling^11^ for the majority of cases and (ii) a quantitative method, the multicriteria decision analysis (MCDA) for difficult or contentious cases, especially when the benefit–risk balance is marginal.^12^ The EMA saw a key role for quantitative modelling in monitoring the benefit–risk balance of a medicinal product post-approval.^13^ However, questions as to how MCDA could be best utilised and the results analysed and communicated have meant that the EMA does not place a requirement for quantitative decision analytic methods. Nevertheless, the EMA accepts the use of such quantitative frameworks via the implementation of an extended ‘Effects Table’^14^—the extension in 2015 of a matrix (initially adopted by EMA regulators in 2009) of the drug’s performance across the effects relevant for licensing purposes—which seeks to communicate transparently the benefit–risk assessment by summarising the key favourable and unfavourable effects that can be attributed to the medicinal product, together with descriptions of their uncertainties, in determining the overall benefit–risk balance.^15^

The US Food and Drug Administration (FDA) began an initiative in 2009 to develop a structured approach for drug benefit–risk assessments—its benefit–risk framework (BRF)^16^—and since 2012 has been legally committed to the phased implementation of ‘a structured risk-benefit assessment framework in the new drug approval process to facilitate the balanced consideration of benefits and risks…and the communication of the benefits and risks of new drugs’.^17^ Concerns over the use of quantitative approaches to benefit–risk assessment are that subjective judgments and assumptions that would inevitably be embodied in their modelling would obscure rather than elucidate the regulator’s thinking.^18^ The BRF thus adopts a qualitative descriptive approach, while acknowledging that quantification of some of its elements is an important part of the decision-making process.^19^

### C. Vaccine Benefit–Risk Analyses: The ADVANCE Project

High-income countries have developed large linked databases, such as the United States CDC Vaccine Safety Datalink project^20^ and the FDA Post-Licensure Rapid Immunization Safety Monitoring system^21^ to address the need for post-marketing monitoring of vaccine safety, and the determination of preliminary estimates of vaccine effectiveness.^22^ In Europe, the European Centre for Disease Prevention and Control sponsored the Vaccine Adverse Event Surveillance and Communication consortium to conduct a distributed case-control study involving databases in Denmark, France, the Netherlands, Sweden, and the UK to evaluate the risk of Guillain–Barré syndrome associated with the H1N1 vaccine.^23^ In the aftermath of the 2009 H1N1 pandemic, the Accelerated Development of Vaccine Benefit-Risk Collaboration in Europe (ADVANCE) project was initiated to create a sustainable pan-European database network for post-market vaccine benefit–risk analyses.^24^ This network was tested in a proof-of-concept study, comparing retrospectively the benefit–risk profiles of whole-cell and acellular pertussis vaccines.^25^

### D*.* Remaining Concerns

While it has been suggested that quantitative decision analysis should complement and extend the FDA’s BRF to better support the appraisal of evidence and improve decision outcomes,^26^ there remains continued reluctance by regulators to adopt formally quantitative approaches to evaluating benefit–risk profiles of medicinal products. This may be due to a desire to maintain a neutral position or a present inability to implement such complex methods.^27^ The expectation is that those benefit–risk assessments published by the newly independent UK Medicines & Healthcare products Regulatory Agency (MHRA) will adhere to the qualitative weighing up of the benefits and risks currently utilised by the EMA.^28^ However, while there has been marked improvement in benefit–risk evaluation, the need for improved communication of benefit–risk evaluation between decision makers and stakeholders remains a key issue.^29^ Poor communication is often a factor that allows controversies surrounding health risks to escalate and opposed groups to become polarised.^30^ Communication strategies to better inform public perceptions of vaccines and to manage inaccurate perceptions of vaccination risks are needed.^31^ This is not merely the role of public health bodies, manufacturers of vaccines and government: multidirectional communication is required.^32^ The extent to which the legal process may have a role to play in such communication in the resolution of vaccine liability disputes is now explored.

## III. VACCINE LIABILITY AND RISK–BENEFIT

### A. Background

In the context of liability for vaccines, courts and tribunals have not been uniformly effective at communicating about risks and benefits. While the primary responsibility for vaccine risk communication lies generally with the government that recommends vaccination,^33^ the legal process also has an important but largely ignored role. This is especially true in the determination of disputes concerning whether vaccines are defective or whether they cause adverse effects to claimants. In the context of COVID-19 vaccines, an opportunity has arisen to reappraise these problems given the importance of the continued availability and supply of vaccines during the pandemic.

While the USA has shifted towards statutory no fault systems for adjudicating vaccine injury claims, the UK retains strict product liability as a potential route to compensation for defective vaccines, notwithstanding the existence of a pre-existing *ex gratia* compensation system, the Vaccine Damage Payments Scheme (VDPS) (under the Vaccine Damage Payments Act 1979), which does not require proof of defectiveness for recovery.^34^ Nonetheless, all these forms of liability require some element of proof of causation between the vaccine and the injury. ^35^ It is submitted that role of scientific evidence remains central to the resolution of vaccine liability disputes, and that a reappraisal of this role is prescient, given the public interest in continued availability and supply of COVID-19 vaccines during the pandemic. The function of this article is to examine this role in the context of determining a vaccine’s defectiveness.

### B. Determining a Vaccine’s Defectiveness

#### 1. Holistic approach to defectiveness: embracing risk–benefit, including the public interest

When vaccines are subject to strict liability under the UK Consumer Protection Act 1987 (CPA), the claimant must establish that the vaccine is defective.^36^ A vaccine is therefore defective if it does not provide the safety that persons generally are entitled to expect, taking all circumstances into account.^37^ An individual’s decision as to whether to accept a vaccine may be partly determined by a weighing up of risk and benefits.^38^

One would conceivably expect that, given a medicinal product ‘will inevitably have some risks attached’,^39^ in determining a vaccine’s defectiveness by taking *all* circumstances into account, ‘a holistic approach… balancing all relevant considerations’,^40^ embracing analysis of the benefits and disadvantages associated with a vaccine (or risk–utility), would be adopted: the expectation of those who take the vaccine will be shaped by that benefit–risk balance. This would be especially relevant in instances of defects concerning the vaccine’s design. For example, even if it were accepted that the measles, mumps and rubella (MMR) vaccine^41^ was capable of having severe side effects, this should not mean that it is automatically to be adjudged defective. The risks would require to be balanced against the serious risks involved in not immunising children and it is highly probable that a court would conclude that the benefits of vaccination outweigh the perceived risks.^42^ A similar argument can be made for the H1N1 pandemic vaccine Pandemrix, given to at least 6 million people in the UK.^43^ While there is compelling epidemiological evidence of an increased risk of narcolepsy following vaccination with Pandemrix, especially in children, ^44^ any court would likely conclude that the considerable benefits of the vaccine would outweigh the small risk of narcolepsy.

In English law, some doubt had been cast on the use of a medicinal product’s risk–benefit profile in determining defectiveness by Burton J in the hepatitis C litigation,^45^ in particular through his assessment of the *travaux préparatoires* to the Product Liability Directive, which he claimed had rejected risk–utility analysis.^46^ However, this was dispelled by the landmark decision of Hickinbottom J (as he then was) in *Wilkes v DePuy International Ltd* in its adoption of a holistic approach to defectiveness, and in its establishment that risk–benefit may be a central part of the question of determining defectiveness in strict product liability for the purposes of the CPA.^47^ In *Wilkes*, Hickinbottom J held that any assessment of a medicinal product’s safety ‘will necessarily require the risks involved in use of that product to be balanced against its potential benefits including its potential utility’.^48^ This risk–benefit balance, together with ‘the ease and extent to which a risk can be eliminated or mitigated’ (avoidability) were potential circumstances in the assessment of a product’s safety.^49^*A fortiori*, specifically in the context of vaccines, Hickinbottom J also opined that a particular medicinal product such as a vaccine ‘may require consideration of a *wider* range of risks and benefits, including the public interest’.^50^ Given the communitarian benefits of vaccines to ‘not only its direct users, but also the population as a whole, as a result of its containment of the disease’,^51^ it suggests that any immunity conferring benefits on both the individual and the community would be relevant to any risk–benefit analysis.

Affirming the role of the risk–benefit calculus in the determination of defectiveness, Mrs Justice Andrews in *Gee v DePuy International*^52^ observed that a wider range of benefits might properly have a bearing on an assessment of defectiveness,^53^ particularly in the context of medicinal products. In her view, since a product’s reasonably expected use was a relevant circumstance in determining defectiveness,^54^ it could not be objectionable for the court to consider the benefits likely to arise from its contemplated use in determining defectiveness.^55^ Accordingly, in discussing counsel’s example of a new chemotherapy drug that had proven advantages over all others on the market, but a rare and serious side effect, she concluded that ‘the additional benefit is plainly a relevant circumstance that would assist in the evaluation of safety by reference to the test set out in s.3 of the Act’.^56^ Here the benefit was the fact that the drug was more ‘effective than other chemotherapy drugs that did not have that side effect’.^57^ The use of the word ‘effective’ here is important, and should be distinguished from that of ‘efficacy’.^58^ A medicinal product is deemed ‘effective’ if it does more good than harm under usual circumstances of healthcare practice, as opposed to efficacy, which is whether a treatment does more good than harm under ideal circumstances, such as a randomised trial.^59^ In the context of vaccines, ‘[e]ffectiveness is how well the vaccine works in the real world’,^60^ and that is clearly relevant to an overall determination of a *wider* range of risks and benefits, including the public interest in the generation of vaccine confidence.

Any lingering uncertainties as to the use of a holistic approach to defectiveness, including the use of a medicinal product’s risk–benefit profile as applied by the High Court decisions in *Wilkes* and *Gee*, were conclusively laid to rest by the Court of Appeal’s endorsement of such an approach in *Bailey v GlaxoSmithKline UK Ltd*.^61^ Arguments against the imposition of the risk–benefit calculus in determining defectiveness in this context continue to be posited, but it is respectfully submitted that they are unconvincing. First, the submission that the benefits of a product can already be taken into consideration by merely considering a medicinal product’s presentation^62^ is misconceived: the risk–benefit profile of a medicinal product cannot be defined merely in terms of the presence or absence of a product’s warning of a side effect. Thus, in *Bailey*^63^ it was noted in the defendant’s case as to the lawful approach to defect concerning the antidepressant Seroxat that ‘any proper comparison between medicines would need to include a comparison of the relative risk/benefit profiles of the medicines being compared, both generally and for the particular Claimant…’.

Secondly, the contention that a vaccine’s benefits may change due to, for instance, subsequent virus mutation^64^ is overstated. The level of safety is the one considered appropriate at the time when the product was supplied.^65^ Any other approach would provide little incentive to improve safety standards in the production of new vaccines. Moreover, those changes in levels of safety due to increased number of viral mutations^66^—an issue confronted in the rollout of the recent COVID-19 vaccines—is precisely the reason why a robust systematic assessment of benefit–risk as a routine adjunct to post-marketing surveillance is so important. To omit this from the court’s determination of defectiveness would be to ignore the iterative approach to benefit–risk analysis, which is crucial to the determination of a vaccine’s safety. There is little doubt now that if a vaccine design defect case were ever to be litigated in the UK, and it concerned a defect in the vaccine’s design, this flexible, holistic approach utilising risk–utility would be used in any determination.^67^

#### 2. Uncertainty as to the role of risk–utility in Europe; a failure in risk communication: NW v Sanofi Pasteur MSD SNC

There has been much debate about the relevance of risk–utility considerations in Europe.^68^ In the context of design defects, the German Supreme Court held in 2009 that a cost–benefit analysis may be appropriate to determine whether a product is defective in design. It ruled that the court should have regard inter alia to ‘the costs of production, the marketability of the alternative design as well as a cost-benefit balance’ and in this respect expressly referred to ‘the risk utility test under US law’.^69^ Although the design defect in question was not in the context of medicinal products (the German Product Liability Act provisions do not apply to pharmaceutical products, which are specifically covered by a strict liability compensation regime in the Medicines Act 1976 (*Arzneimittelgesetz* 1976) (AMG)), the case indicates that the notion of risk–benefit is relevant to liability for design defects.^70^*A fortiori*, in the context of pharmaceuticals, the AMG provides that if a pharmaceutical product’s benefits outweigh its risks, it cannot have a design defect.^71^ Thus in holding that the hepatitis B vaccine was not defective, the risk–benefit balance was applied in one of the few cases on defectiveness of vaccinations under the AMG where a child had turned blind subsequent to vaccination.^72^

However, it is in France that there has been the greatest controversy. While the risk–utility approach to defectiveness has been adopted by some French courts in the context of medicinal products,^73^ the French Cour de cassation^74^ overruled the use of a general risk–utility analysis in the context of the hepatitis B vaccination litigation, alleging that the vaccine caused multiple sclerosis (MS). Though the Cour d’appel de Versailles^75^ had ruled that the temporal proximity between the hepatitis B vaccination and the manifestation of the demyelinating disease, in the absence of any other known cause for the disease, allowed a presumption that the vaccine had caused the claimant’s injury, the appellate court rejected the claim against the vaccine producer, by determining, utili**s**ing a risk–benefit analysis, that the vaccine was not defective. However, the decision on defectiveness was subsequently quashed by the Cour de cassation, which held that the Cour d’appel de Versailles had failed to provide a legal basis for its decision in respect of the vaccine not being defective. It held that Cour d’appel de Versailles should have checked if the elements, on the basis of which causation had been presumed, did not also allow a presumption that the vaccine was defective. The Cour de cassation suggested that defectiveness could be assessed on a case-by-case basis, independently from a ‘general’ risk–benefit analysis, taking into account the specific considerations of the product.^76^ In rejecting the risk–benefit analysis as a general test, the Cour de cassation omitted detail of any alternative test, merely noting some elements that could be used to establish defectiveness, on a case-by-case basis.^77^ It was on a second appeal brought by the claimants that the Cour de cassation decided to stay the proceedings and refer the matter to the Court of Justice of the European Union (CJEU).^78^

On the reference to the CJEU, *NW and others v Sanofi Pasteur MSD SNC*,^79^ the Court examined the issue of how to determine liability under the Product Liability Directive, where there is an absence of scientific evidence establishing that a product (in this case, the hepatitis B vaccine) was capable of causing damage (MS), and merely circumstantial evidence.^80^ Instead of clearly distinguishing between the requirements of establishing defectiveness under Article 6 and causation under Article 4, the CJEU proceeded to conflate the separate issues of causation and defectiveness. It did so by concluding that, notwithstanding the absence of scientific consensus concerning a causal link between a vaccine and the occurrence of a disease, certain factual evidence constituting ‘serious, specific and consistent presumptions’ that could support a finding of causation could be relied on to prove that the vaccine was defective. ^81^ As a result, the CJEU gave its unwarranted blessing to the use of prima facie facts that could support a finding of causation to find the product defective.

The factors relied on by the court to constitute such ‘serious, specific and consistent presumptions’^82^ in determining causation bear little relationship to the kinds of factors that a court must take into account in determining whether a product is defective under Article 6, including the risk–benefit ratio of the product. That this is an erroneous conflation of causation and defectiveness by the CJEU is clear from a cursory reading of the Directive: the concepts of defect under Article 6 and causation under Article 4 are distinct.^83^ The conflation of the two concepts also sits uneasily with the CJEU’s own admonition against national courts applying evidentiary rules in such a manner that where types of evidence are presented together, an immediate and automatic presumption would operate of there being a defect in a product and/or a causal link between the defect and damage.^84^ Despite the requirement under Article 6 to take ‘all circumstances’ into account in determining defectiveness, the CJEU took no account of the holistic approach adopted by the English High Court in *Wilkes v DuPuy International Ltd*. ^85^ In *Wilkes*, Hickinbottom J (as he then was) noted that in determining whether a hip prosthesis was defective under the CPA, relevant circumstances in the assessment of the product’s safety include the risk–benefit profile of the product,^86^ the cost of the product,^87^‘the ease and extent to which a risk can be eliminated or mitigated’ (avoidability),^88^ compliance with standards,^89^ regulatory approval,^90^ and the warnings and information provided with the product.^91^ As has been previously noted, Hickinbottom J also observed that a particular medicinal product such as a vaccine may require consideration of a wider range of risks and benefits, including the public interest.^92^

While the CJEU was silent on the matter of risk–benefit, Advocate General Bobek did expressly raise the matter in his Opinion, and stated that he demurred with the proposition that the notion of defect involved ‘[a] broader assessment of the cost/benefits of the product … going beyond the concrete case’.^93^ He proceeded to opine that the test of defectiveness

essentially refers to baseline expectations of the product under normal conditions of use. It does not mean that where the product is used normally and causes serious harm in an individual case, that a conclusion of defectiveness necessarily requires a balancing of the costs and benefits of the product.^94^

In his view, such an approach would result in the court ‘creating (or at least boldly deducing) new conditions of liability’.^95^ However, in the English High Court decision of *Gee v DePuy International*,^96^ the observations of the Advocate General were regarded as confined to the context of submissions made by the respondents that ‘a broader assessment of costs and benefits is *always* required’, but that was ‘quite different from suggesting that they could *never* be considered relevant’.^97^ Nothing could be drawn from the observations of the Advocate General (or the silence of the CJEU) in *Sanofi Pasteur* to support the submission that risk–benefit is irrelevant.^98^

The CJEU’s approach to defectiveness has been subject to criticism. Professor Borghetti condemns the approach of the CJEU as one that seems to invite the ‘deplorable result’ of decisions of lower courts that would regard the hepatitis B vaccine as defective by a presumption that it causes a severe demyelinating disease in a given case, notwithstanding the vaccine’s overall positive risk–benefit ratio, and an absence of anything wrong with the batches used by the claimant.^99^ It is submitted that the tacit approach of the CJEU on risk–benefit is unrealistic in the context of vaccines, where a balancing of risk and benefit is an unavoidable element in reflecting a holistic approach to defectiveness and the need for all circumstances to be taken into consideration in making such a determination.^100^ Moreover, by conflation of the separate issues of defectiveness and causation and omitting any discussion of the risk–benefit profile of the vaccine, the CJEU undermines the scientific basis for the determination of a vaccine’s safety and in so doing may increase the potential for vaccine hesitancy.^101^ Thus in the context of the COVID-19 pandemic, global surveys on the public’s attitude to taking a coronavirus jab show that concern about side effects is the principal reason for not wanting to have the vaccine,^102^ but more specifically there remains a belief amongst many that the side effects and potential risk of the vaccine are worse than the disease itself.^103^ This suggests a failure to communicate effectively to the public on the question of risk–benefit, whose legitimate expectations on vaccine safety are shaped by such a calculus. In turn, the risk of vaccine hesitancy is potentially increased by the failure to consider public interest or policy factors in a wider analysis of risk–benefit considerations,^104^ such as vaccines furthering the public interest in health and safety through vaccine confidence and the overall communitarian benefit to the public of herd immunity through a vaccine.

#### 3. Lessons from the USA: risk–benefit and Comment k of section 402A

Advocation of strict liability in tort for vaccine-induced injuries had its origins in the Cutter Incident.^105^ A 1955 *Yale Law Journal* article on the matter^106^ foretold the ‘nationwide trend towards strict products liability’ of the 1960s^107^ by recommending ‘strict liability in *tort* for all injuries directly resulting from the infective vaccine [manufacturers] produce’.^108^ However, it was a California appellate court that ‘opened the door’^109^ to strict product liability claims for vaccine—induced adverse events. In *Gottsdanker v Cutter Laboratories*,^110^ it was claimed that two children contracted poliomyelitis shortly after being inoculated with Salk polio vaccine.^111^ The essence of the claim was not that the vaccine failed to prevent polio (a question of efficacy) but that the vaccine had caused the disease (a question of vaccine safety).^112^ Three causes of action were submitted to a jury, one in negligence and two for breach of implied warranty. A jury had concluded that Cutter was not negligent but that they were liable under breach of implied warranty theory.^113^ The appellate court affirmed the jury’s verdict on the warranty theory, though concluding that it was unnecessary to consider the appeal on the negligence issue.^114^ Upholding the distinction between liability for vaccine safety and non-liability for vaccine efficacy,^115^ was in line with the ‘nationwide trend towards strict products liability’ of the 1960s.^116^ While vaccines were initially unaffected by *Gottsdanker*, several cases emerged addressing strict product liability and vaccine-related injuries, the earliest concerning the Sabin live attenuated oral polio vaccine (OPV). Decisions have been heavily influenced by the *Restatement, Second, Torts* 402A (1965). § 402A does not distinguish between prescription and other products and hence liability is imposed where the product is ‘in a defective condition unreasonably dangerous to the user or consumer’ even where ‘the seller has exercised all possible care in the preparation and sale of his product’.^117^

In the context of vaccines, as with other prescription drugs,^118^ there are inherent difficulties in applying strict tort liability. For example, in *Kearl v Lederle Laboratories,*^119^ which involved an OPV, the appellate court noted that the application of a strict liability standard might cause delay in the marketing of pharmaceutical products and could deter their research, manufacturing, and marketing.^120^ The court added that while strict liability was ‘socially beneficial in the vast majority of products cases’, ‘it might not be appropriate with regard to some special products that are extremely beneficial to society and yet pose an inherent and substantial risk that is unavoidable at the time of distribution’.^121^ Comment *k* of 402A attempts to address such concerns directly.^122^ In acknowledging that many pharmaceutical products, including prescription products, ‘are quite incapable of being made safe for their intended and ordinary use’, the comment establishes that both the marketing and the use of such products are fully justified, notwithstanding the unavoidable high degree of risk that they involve. Such a product, Comment *k* explains, ‘properly prepared, and accompanied by proper directions and warnings, is not defective nor is it unreasonably dangerous’. Comment *k* notes the ‘outstanding example’ of an unavoidably unsafe product as being:

the vaccine for the Pasteur treatment of rabies, which not uncommonly leads to very serious and damaging consequences when it is injected. Since the disease itself invariably leads to a dreadful death, both the marketing and the use of the vaccine are fully justified, notwithstanding the unavoidable high degree of risk which they involve.

Accordingly, vaccines were at an early stage of the development of strict liability posited as being products that could be classed as unavoidably unsafe, and, if ‘properly prepared, and accompanied by proper directions and warnings’, deemed neither defective nor unreasonably dangerous. This no-design liability rule tended to be *linked* to a risk–utility analysis. In *Reyes v Wyeth Labs*,^123^ the Sabin live polio vaccine was unavoidably unsafe since the live poliomyelitis virus, the essence of the vaccine, always presented the danger of causing poliomyelitis. Utilising its two-step analysis to determine that the product was not unreasonably dangerous *per se*, the utility of the vaccine in preventing paralysis was held by the Fifth Circuit Federal Court of Appeals to far outweigh the ‘statistically minuscule’ risk that the vaccine could cause polio.^124^

By the mid-1980s, the use of Comment *k* as a no-design liability rule had become increasingly undermined by requiring drug and vaccine manufacturers to prove their product was unavoidably unsafe.^125^ A more demanding approach to the Comment *k* exemption was adopted by the *Kearl* court, with risk–benefit as its springboard. While agreeing in principle with exemption of unavoidably dangerous products adumbrated in Comment *k*, it held that the decision to trigger this exemption posed a mixed question of law and fact and required a risk–benefit analysis—to be carried out at a ‘mini trial’ before a judge—as a *prerequisite* to determining whether Comment *k* exempted the vaccine from strict liability.^126^ The trial court would decide to exempt a product from strict liability only after first taking evidence outside the jury’s presence as to:

(1) whether, when distributed, the product was intended to confer an exceptionally important benefit that made its availability highly desirable; (2) whether the then-existing risk posed by the product both was “substantial” and “unavoidable”; and (3) whether the interest in availability (again measured at the time of distribution) outweighs the interest in promoting enhanced accountability through strict liability design defect review.^127^

However, an additional element to determining whether the risk was ‘unavoidable’ required the consideration at the time of distribution of the availability of any alternative product that would have ‘*as effectively* accomplished’ the subject product’s ‘*full intended purpose*’ with a lesser risk.^128^ In reviewing designs to determine defectiveness, courts began to hold drug and vaccine manufacturers liable for failing to adopt a safer design.^129^ At first glance, it seemed acceptable under a risk–utility analysis that if another vaccine manufacturer had previously marketed an FDA-approved vaccine that had equal or greater benefits but fewer risks than the FDA-approved vaccine in issue, liability could be imposed on the manufacturer of the vaccine with the greater risk that harmed the plaintiff.^130^ However, increased accountability of vaccine manufacturers reached its zenith with liability for failing to develop and make available a safer vaccine that had received no FDA approval in *Toner v Lederle Laboratories*.^131^ In *Toner*, the principle thrust of the design claim concerned Lederle’s knowledge of and failure to develop a safer, less toxic fractionated cell pertussis vaccination and seek FDA certification of it, rather than a whole cell vaccine.^132^ While the jury rejected a claim in strict liability, it found Lederle negligent.^133^ Affirming the jury’s verdict that Lederle’s design was negligent, the Idaho Supreme Court held that Comment *k* did not shield sellers of products from negligence claims for design defects.^134^ The court observed that Comment *k’*s application depended on a ‘balancing between risks and benefits…similar to those involved in a negligence claim’,^135^ as well as the ‘availability of a feasible alternative design’.^136^ The court had nonetheless noted that the FDA had refused to license any fractionated cell vaccine, and that sale of such a vaccine would constitute a criminal offence under the Food, Drug and Cosmetic Act.^137^ This ‘downright baffling’^138^ opinion would require a jury to determine that the FDA would have approved such a vaccine had it been developed, which would have amounted to no more than guesswork.^139^ As had been presaged by the Institute of Medicine at the time of the jury verdict, juries ‘could easily become the de facto regulators of immunization practices in the United States’,^140^ and unfavourable jury verdicts could effectively stop production of a vaccine.^141^

Nonetheless, the significance of Comment *k* in the context of vaccines in the USA has now somewhat evaporated, particularly in the context of design defect claims. Consequent upon concern in the 1980s around vaccines against DPT, which were blamed for disabilities and developmental delays in children, a ‘massive increase in vaccine-related tort litigation’^142^ was generated, culminating in more than 200 suits each year. This resulted in a destabilisation of the DPT vaccine market, and the withdrawal of two out of three of the American domestic manufacturers.^143^ The litigation costs, the rise in prices of vaccines, and the instability and unpredictability of the childhood vaccine market, together with increasing concern over the uncertainties of obtaining compensation for injuries due to vaccines, resulted in the passing by Congress of the National Childhood Vaccine Injury Compensation Act of 1986 (NCVIA).^144^ The legislative history of the Vaccine Act indicated Congress’ express intent to codify the principle in Comment *k* regarding unavoidably unsafe products.^145^However, the question arose as to whether Congress intended to remove design defect litigation from the tort system.

There is a broad pre-emption of state tort claims in 42 USC 300aa-22. Paragraph (b)(1) provides:

No vaccine manufacturer shall be liable in a civil action for damages arising from a vaccine-related injury or death associated with the administration of a vaccine after October1, 1988, if the injury or death resulted from side effects that were unavoidable even though the vaccine was properly prepared and was accompanied by proper directions and warnings’.^146^

Given Congress’ expressed intent to codify the principle in Comment *k*, it was argued by the petitioners in *Bruesewitz v Wyeth LLC* that Comment *k* was invoked by the use of the term ‘unavoidable’ in 300aa-22(b)(1) as a term of art,^147^ but this was rejected by the Supreme Court.^148^ The Court focused on the text of 300aa-22(b)(1), barring civil action liability where the injury or death resulted from side effects that were unavoidable, ‘*even though* the vaccine was properly prepared and was accompanied by proper directions and warnings’. The Supreme Court found that the words ‘even though’ clarified the words that proceeded it, and that the statute established as a complete defence unavoidability (given safe manufacture and warning) with respect to the particular design.^149^ It held that the 1986 Act^150^ expressly preempts all state law design defect claims against manufacturers for injury or death caused by vaccine side effects.^151^ State law design defect vaccine injury litigation that concerns vaccines listed on the Vaccine Injury Table^152^ is thus barred, yet the Court’s conclusion appears inherently suspect: there is much to be said of the conclusion in the dissent that the majority based their decision on policy preference over legislative intent.^153^

In support for this view is the fact that Congress *did* intend to incorporate Comment *k* in the NCVIA,^154^ that the failure to mention design defects in the Act or FDA regulations did *not* mean that Congress intended to remove design defect litigation from the tort system,^155^ and that the NCVIA, while providing the means to develop improved designs and to compensate for inflicted injuries,^156^ imposes no *duty* on vaccine manufacturers to improve the design of their vaccines to account for advances in science and technology.^157^ There is also no FDA requirement to condition approval on a vaccine being the most optimally designed among reasonably available alternatives or to ensure that licensed vaccines keep pace with scientific and technological advances.^158^ As the powerful dissent noted, the function of ensuring vaccines are optimally designed in the light of such advances has been traditionally left to states through liability for design defects, with state product liability law acting as a complementary form of drug regulation.^159^

It has thus been submitted that there should be a narrow application of the scope of the preemption doctrine in the case of pharmaceuticals, in order to preserve the tort system as a complement to more formal regulatory responses to uncertainty surrounding risk information.^160^ Nevertheless, the argument remains that allowing design defect litigation where ‘the universe of alternative designs …[is] limited only by an expert’s imagination’^161^could defeat the *quid pro quo* of the Act, which allows manufacturers to fund a compensation programme for vaccine injuries in exchange for avoiding costly tort litigation,^162^ and might ‘potentially jeopardise market stability and vaccine availability’.^163^ The possibility of a claim under state law remains—and there are gaps occasionally found in the NCVIA^164^—but those plaintiffs seeking to utilise state tort law would need to bring claims not pre-empted by the broad provision in 42 USC § 300aa-22.^165^

Despite the erosion of Comment *k’*s significance in liability for vaccine defects, and criticism of both macro^166^ and micro^167^ risk–utility balancing of drug designs in favour of an ‘individualized, nonaggregative approach to benefit-risk analysis’ for prescription drugs, ^168^ it is clear that the adoption of a risk–benefit analysis as a *prerequisite* to determining whether Comment *k* exempted the vaccine from strict liability played a key role in the determination of strict product liability claims prior to the NCVIA. Under a risk–benefit analysis, strict liability could be imposed on the manufacturer of a vaccine that harmed the plaintiff if another vaccine manufacturer had marketed an FDA-approved vaccine that had *equal or greater benefits but fewer risks* than the FDA-approved vaccine in question.^169^ Moreover, the purpose of Comment *k*, in its barring of strict liability when such a liability ‘would undermine the public interest in product safety’^170^ suggests that public interest or policy factors, such as vaccines furthering the public interest in health and safety through vaccine confidence and the overall communitarian benefit to the public of herd immunity through a vaccine, should indeed be factored into the determination of defectiveness in a strict product liability regime that has no equivalence to the NCVIA in its pre-emption of design defect claims against manufacturers.^171^

#### 4. Risk–benefit in determining liability for COVID-19 vaccines

The use of risk–benefit in determining defectiveness under the CPA has the potential to play an important part in assessing liability for a vaccine against COVID-19. In so doing it can be viewed as part of a communication strategy through judicial decision-making to better inform public perception of vaccines.^172^ While the preferred route to enable deployment of a new vaccine is through the usual marketing authorisation (product licensing) process, the Department of Health and Social Care indicated in 2020 that if there was a ‘compelling case, on public health grounds, for using a vaccine before it is given a product licence’, the Joint Committee on Vaccination and Immunisation may advise the UK government to use a tested, unlicensed vaccine against COVID-19.^173^ Approval of the supply of a Covid-19 vaccine can be taken under Regulation 174 of the Human Medicines Regulations, which enables the rapid temporary approval of medicinal products to respond to significant public health issues, including pandemics.^174^ The decision to approve the first COVID-19 vaccine for the UK—the mRNA vaccine BNT162b2 developed by Pfizer/BioNtech—was taken on this basis,^175^ as was the subsequent approval of both the viral vector ChAdOx1 nCov-19 vaccine, developed by Oxford University/Astro-Zeneca ^176^ and the mRNA Moderna Covid-19 vaccine.^177^

Article 5(3) of the EU Medicines Directive^178^ requires Member States to lay down provisions to ensure that marketing authorisation holders, manufacturers, and health professionals are not subject to civil or administrative liability for any consequences resulting from the use of a medicinal product otherwise than for its authorised indications, or from the use of an unauthorised medicinal product, when such use is by the licensing authority in response to, *inter alia*, the spread of pathogenic agents. This requirement has been transposed into UK law,^179^ resulting in an exclusion of civil liability claims against manufacturers, marketing authorisation holders, and health professionals under contract, tort, and breach of statutory duty. However, immunity is not absolute. In accordance with Article 5(4) of EU Medicines Directive, UK law preserves the application of strict product liability under section 2 of the CPA.^180^ Accordingly, when a tested, unlicensed vaccine against COVID-19 is used, liability will be channelled towards a strict liability cause of action under the CPA.^181^ Determination of whether a COVID-19 vaccine is defective will therefore be a central issue.^182^

Given the rise in vaccine hesitancy, the role of public interest in the consideration of a wider range of risks and benefits as suggested in *Wilkes* *v DePuy International Ltd*^183^ will necessarily be important in determining defectiveness in the context of a SARS-Cov-2 vaccine under the CPA. It is self-evident that vaccines designed to address a *global* health problem such as the COVID-19 pandemic, which benefit the wider community and not just the individual, necessitate a *broader* approach to risk–benefit, which includes the public interest. Here, these wider public interest concerns should reflect the need for vaccine confidence and the overall communitarian benefit to the public of herd immunity through a vaccine.^184^

Whilst by no means impossible, establishing a vaccine’s defectiveness will remain an extremely difficult hurdle to overcome. As we have previously noted, under a risk–benefit analysis, if another vaccine manufacturer had marketed an approved vaccine that had *equal or greater benefits but fewer risks* than the FDA-approved vaccine in issue, liability could be imposed on the manufacturer of the vaccine with the greater risk that harmed the plaintiff.

Echoes of this strategy would seem to have been belatedly adopted by the claimants in *Bailey v GlaxoSmithKline UK Ltd*, when they argued at trial that Seroxat had no particular benefits relative to other drugs in the appropriate comparative group and that a ‘level playing field’ should be assumed with regard to the benefits and risk associated with Seroxat and its comparator drugs, save for discontinuation symptoms, of which they claimed Seroxat was the ‘worst in class’.^185^ However, the Court of Appeal rejected this argument on the basis that this was beyond the scope of pleadings.^186^ Given the defendant's case that a holistic assessment of defectiveness should include ‘the relative risk/benefit profiles of the medicines being compared’, ^187^ it was of no surprise that the defendant had never conceded that Seroxat had no particular relative benefits.^188^ It is highly unlikely that the manufacturers of a COVID-19 vaccine would concede that their product had no particular relative benefits. Nevertheless, the use of the relative risk–benefit profiles of all COVID-19 vaccines which have received temporary authorisation in the UK could clearly be relevant to any holistic assessment of defectiveness. Moreover, as with other medicinal products, the risk–benefit profile of vaccines may change with the age group of those to whom they are administered. Concerns have arisen about potential adverse effects of the COVID-19 vaccines, including the rare risk of blood clots with the COVID-19 Vaccine AstraZeneca, with a higher incidence in younger adults.^189^ If the younger adult was given the vaccine and was perfectly healthy, the balance could possibly come down in favour finding the vaccine defective.^190^

A balancing of the benefits and disadvantages associated with the product, together with the cost and practicability of producing an equivalent product without such disadvantages was approved in *Wilkes v DePuy International Ltd*, where Hickinbottom J held that both the cost and the ‘practicability of producing a product of risk–benefit equivalence’ were both potentially relevant circumstances in the assessment of a product’s safety.^191^ This will be difficult to establish, as has previously been seen in comparing the Sabin OPV (using an attenuated form of the viral agent) with the Salk inactivated polio vaccine (IPV) (using a killed virus).^192^ Given the requirement for speed in the context of limited supplies of COVID-19 vaccines, any effort to compare them will need to take into consideration ‘not only their reported effectiveness, but also supplies, costs, the logistics of deploying them, the durability of the protection they offer and their ability to fend off emerging viral variants’.^193^ Nonetheless, with unprecedented data emerging from the roll-out of these vaccines, and given the marked improvement in benefit–risk evaluation, and the determination of preliminary estimates of vaccine effectiveness,^194^ this does not appear insurmountable. Since a vaccine ‘may require consideration of a *wider* range of risks and benefits, including the public interest’,^195^ it is submitted that any immunity conferring benefits on both the individual and the community of the COVID-19 vaccines would be crucial to any determination of defectiveness. As has been previously noted,^196^ the need to develop communication strategies that frame the benefits of vaccination and the risks of not vaccinating to better inform public perception of vaccines has been highlighted by the ADVANCE project.^197^ It is submitted that the court’s role in determining the defectiveness of vaccines using a holistic approach, including risk–benefit, may help manage inaccurate perceptions of vaccination risks by being a part of such a communication strategy through judicial decision-making to better inform public perception of vaccines.

## IV. CONCLUSION

A plethora of vaccines has emerged as the best therapeutic armamentarium to address the COVID-19 pandemic. As new vaccines have received temporary authorisation under an unprecedented timescale, the messaging from government and public health agencies has been about the need for the benefits of vaccines to outweigh the risks. Concerns have arisen about potential adverse effects of the COVID-19 vaccines, including the rare risk of blood clots with the COVID-19 Vaccine AstraZeneca. The issue of vaccine liability for adverse effects, whilst overshadowed by the pandemic, is in fact one of immediate importance.

This article has sought to argue that in the resolution of controversies about vaccine liability, a holistic, flexible approach to defectiveness embracing risk–benefit allows consideration of the overwhelming public interest derived from the continued availability and supply of vaccines, as well as immunity conferring benefits on both the individual and the community. Any doubts as to the use of a holistic approach to defectiveness, including the use of a vaccine’s risk–benefit profile, have been conclusively laid to rest by the Court of Appeal’s endorsement of such an approach in *Bailey v GlaxoSmithKline UK Ltd*. The implications for vaccine liability are considerable. If a vaccine design defect case was ever to be litigated in the UK, and it concerned a defect in the vaccine’s design, this flexible, holistic approach utilising risk–utility would likely be used, requiring consideration of a wider range of risks and benefits than other medicinal products.

Unlike the UK, the debate about the relevance of risk–utility considerations in Europe continues: the CJEU’s conflation of the separate issues of defectiveness and causation and omission of any discussion of the risk–benefit profile of the vaccine in *Sanofi Pasteur* undermines the scientific basis for the determination of a vaccine’s safety and in so doing may increase the potential for vaccine hesitancy. Despite the erosion of Comment *k’*s significance in liability for vaccine defects in the USA, the adoption of a risk–benefit analysis as a prerequisite to determining whether Comment *k* exempted the vaccine from strict liability played a key role in the determination of strict product liability claims prior to the NCVIA.

In short, the overall risks and benefits of vaccines as part of a holistic approach to determining defectiveness should not be eschewed by product liability lawyers. To omit risk–benefit from a judicial determination of defectiveness would be to ignore the iterative approach to benefit–risk analysis which is crucial to the determination of a vaccine’s safety and may unwittingly weaken vaccine confidence. The use of risk–benefit in determining defectiveness under the CPA has the potential to play an important part in assessing liability for a vaccine against COVID-19. In so doing it can be viewed as part of a communication strategy through judicial decision-making to better inform public perception of vaccines. If cases do emerge concerning the liability of a COVID-19 vaccine, immunity conferring benefits on both the individual and the community of the COVID-19 vaccines should be relevant in any determination of defectiveness. Such a holistic, flexible approach to defectiveness embracing risk–benefit can be used effectively to determine the entitled safety of a vaccine and may help to mitigate against the dangers of weakening confidence in the public’s vaccine uptake.

*Conflict of interest statement*. None declared.

## Footnotes

1 Sir Jeremy Farrer, ‘Forward’, in *Wellcome Global Monitor – First Wave Findings* (Gallup 2019) 4 <https://wellcome.org/reports/wellcome-global-monitor/2018> accessed 15 February 2021.

2 U Heininger, ‘A Risk-Benefit Analysis of Vaccination’ (2009) 27 Vaccine G9.

3
*Loveday v Renton* [1990] Med LR 117, 122, 182; Heininger, ibid G9–G12.

4 MZ Dudley and others, ‘The State of Vaccine Safety Science: Systematic Reviews of the Evidence’ (2020) 20 Lancet Infectious Diseases e80.

5 Directive 2001/83/EC [2001] OJ L311/67, art 26(1)(b) as amended.

6 ibid, art 1(28) and (28a).

7 Human Medicines Regulations 2012 SI 2012/1916, reg 58(4)(b).

8 Food and Drug Administration, ‘Structured Approach to Benefit-Risk Assessment in Drug Regulatory Decision-Making: Draft PDUFA V Implementation Plan-Fiscal Years 2013-2017’ (US Department of Health and Human Services 2013) 1.

9 MD Rawlins, ‘Trading Risk for Benefit’ in RD Mann (ed) *Risk and Consent to Risk in Medicine* (The Parthenon Publishing 1989) 193–201.

10 J Juhaeri, ‘Benefit-Risk Evaluation: The Past, Present and Future’ (2019) 10 Therapeutic Advances in Drug Safety 1.

11 See, further, MGM Hunink and others, *Decision Making in Health and Medicine: Integrating Evidence and Values* (2nd edn, Cambridge University Press 2014) 5–26.

12 EMA, Benefit-Risk Methodology Project Work Package 4 Report: Benefit-Risk Tools and Processes, 9 May 2012 EMA/297405/2012 3.

13 ibid.

14 A Angelis and LD Phillips, ‘Advancing Structured Decision-Making in Drug Regulation at the FDA and EMA’ (2021) 87 British Journal of Clinical Pharmacology 395, 403.

15 EMA, <Co>Rapporteur day <60*><80> critical assessment report, 2018. The current ET guidance is embodied in the EMA’s 80 Day Guidance document: EMA, Guidance document on the content of the Rapporteur day critical assessment report (EMA, 2017) 53–66.

16 Food and Drug Administration (n 8) 1, 3–8.

17 Federal Food Drug and Cosmetic Act (FD&C Act), 505(d) (21 USC 355(d)), as amended by 905 of the Food and Drug Administration Safety and Innovation Act (FDASIA), Public Law 112-144–July 9, 2012.

18 Food and Drug Administration (n 8) 4.

19 ibid.

20 CDC, 'Vaccine Safety Datalink (VSD)', <https://www.cdc.gov/vaccinesafety/ensuringsafety/monitoring/vsd/index.html> accessed 30 December 2021.

21 M Nguyen and others, ‘The Food and Drug Administration’s Post-licensure Rapid Immunization Safety Monitoring Program: Strengthening the Federal Vaccine Safety Enterprise’ (2012) 21(SI) Pharmacoepidemiology and Drug Safety 291 (created in 2009 as a response to the need to monitor the safety of the H1N1 influenza vaccine).

22 F DeStefano and others, ‘ADVANCE: The Promises, Pitfalls, and Future Prospects of a European Distributed Data Network for Immunization Surveillance and Research’ (Editorial) (2020) 38 Vaccine v.

23 J Dieleman and others ‘Guillain Barré Syndrome and Adjuvanted Pandemic Influenza A (H1N1) 2009 Vaccine: Multinational Case-Control Study in Europe’ (2011) 343 British Medical Journal d3908.

24 Innovative Medicines Initiative, *Advance,* <https://www.imi.europa.eu/projects-results/project-factsheets/advance> accessed 30 December 2021.

25 DeStefano and others (n 22) v, vi.

26 Angelis and Phillips (n 14).

27 J Juhaeri, ‘Benefit-Risk Evaluation: The Past, Present and Future’ (2019) 10 Therapeutic Advances in Drug Safety 1, 5. It has been conceded that regulatory agencies would need to develop competence in interpreting MCDA models for their validity: Angelis and Phillips (n 14).

28 Benefit–risk methodology, European Medicines Agency (europa.eu) <https://www.ema.europa.eu/en/about-us/support-research/benefit-risk-methodology> accessed 27 February 2020.

29 Juhaeri (n 27).

30 P Bennett and others, ‘Understanding Public Responses to Risk: Policy and Practice’ in P Bennett and others (eds), *Risk Communication and Public Health* (2nd edn, OUP 2010) 3.

31 See the discussion by the ADVANCE project of the need to develop communication strategies to better inform public perception of vaccines in E Karafillakis and HJ Larson, ‘The Benefit of the Doubt or Doubts Over Benefits? A Systematic Literature Review of Perceived Risks of Vaccines in European Populations’ (2017) 35 Vaccine 4840, 4846, 4847.

32 See R Cassidy, ‘Risk Communication in the British Pertussis and MMR Vaccine Controversies’ in P Bennett and others (eds), *Risk Communication and Public Health* (2nd edn, OUP 2010) 142.

33 LK Ball and others, ‘Risky Business: Challenges in Vaccine Risk Communication’ (1998) 101 Pediatrics 453, 457.

34 It should be noted that while proof of defectiveness is not required under the VDPS, success rates are extremely low: since its inception, there has been 6,474 claims against the VDPS, of which 946 resulted in awards, a success rate of 14.6%. By far, the majority of claims to the VDPS that have been disallowed were on the basis that the vaccination did not cause the disability (a total of 4,331 claims, amounting to 79%): FOI2020/76078, 11 December 2020; FOI2021/03388, 1 February 2021. The VDPS has been extended to those vaccinated against the COVID-19 virus (Vaccine Damage Payments (Specified Disease) Order 2020, SI 2020/1411, art 2), and the conditions of entitlement have been modified to extend the 1979 Act to those vaccinated against the COVID-19 virus at a time when they were 18 years or older (ibid, art 3). To have not extended the VDPS in this way could have had an effect on vaccine confidence by a failure to treat COVID-19 vaccines in the same way as other national vaccine programmes (‘Expansion of the Vaccine Damage Payments Scheme (VDPS) for COVID-19’, IA No 9564, 2 December 2020, 2, 12).

35 The issue of proof of causation between vaccines and injury and vaccine hesitancy is equally controversial and is addressed in a separate paper.

36 Consumer Protection Act 1987, s 3; implementing Dir 85/374/EEC on the approximation of the laws, regulations, and administrative provisions of the Member States concerning liability for defective products [1985] OJ L210/29, art 6. Hereafter, ‘the Product Liability Directive’.

37 ibid, s 3(1) and (2).

38 D Freeman and others, ‘COVID-19 Vaccine Hesitancy in the UK: The Oxford Coronavirus Explanations, Attitudes, and Narratives Survey (Oceans)’ (2021) II Psychological Medicine 1.

39
*Wilkes v DePuy International Ltd* [2016] EWHC 3096 (QB), [2018] QB, [82] (Hickinbottom J).

40 See *Wilkes* (n 39), [2018] QB, [78]. The adoption of the philosophical adjective ‘holistic’ (explicable by reference to the whole) is apposite in that determining a medicinal product’s safety requires ‘a holistic approach that calls for an integrated assessment of the clinical and laboratory adverse effects associated with the product’: ibid, [14], citing C Gibson and others, ‘Product Liability for Medicinal Products’ in M Powers and A Barton (eds), *Clinical Negligence* (5th edn, Bloomsbury Professional 2015) 13.29.

41 See also the vaccine for the Pasteur treatment of rabies, where the benefits of the vaccine accompanied by adequate warnings would outweigh any known but unavoidable risks: J Stapleton, *Product Liability* (Butterworths 2004) 261, 262.

42 D Fairgrieve and R Goldberg, *Product Liability* (3rd edn, OUP 2020), 10.102. The same could be said for other childhood vaccines, such as the diphtheria, pertussis and tetanus (DPT) vaccine. There remains ‘today no “perfect" or reaction-free childhood vaccine on the market’: See HR Rep No 99-908, 6.

43 MHRA, 'Pandemic vaccines and antivirals: safety review', 11 December 2014, <https://www.gov.uk/drug-safety-update/pandemic-vaccines-and-antivirals-safety-review> accessed 13 April 2021.

44 J Stowe and others, ‘Reassessment of the Risk of Narcolepsy in Children in England 8 Years after Receipt of the AS03-Adjuvanted H1N1 Pandemic Vaccine: A Case-Coverage Study’ (2020) 17(9) PLoS Medicine e1003225; TO Sarkanen and others, ‘Incidence of Narcolepsy after H1N1 Influenza and Vaccinations: Systematic Review and Meta-Analysis’ (2019) 38 Sleep Medicine Reviews 177.

45
*A v National Blood Authority* [2001] 3 All ER 289, [36], [68] and [71].

46 ibid, [35], [43].

47
*Wilkes* (n 39) , [2018] QB, [67], [78]. The holistic approach to defectiveness has now been upheld in several cases. See the Court of Appeal decision in *Bailey v GlaxoSmithKline UK Ltd* [2019] EWCA Civ 1924 [8]. See also *Gee v DePuy International Ltd* [2018] EWHC 1208 (QB) [143] and the Scottish approval of Hickinbottom J’s holistic approach by the Outer House of the Court of Session in *AH v Greater Glasgow Health Board* [2018] CSOH 57, 2018 SLT 535 [114] (Lord Boyd of Duncansby).

48 ibid, [82].

49 ibid, [82], [89].

50 ibid, [66] (emphasis added). See also *Gee* (n 47) [152]; J Stapleton, ‘Liability for Drugs in the US and EU: Rhetoric and Reality’ (2007) 26 Review of Litigation 991, 1020 (the approach to undertake in determining a claim for defectively designed medicinal products is to utilise a cost–benefit approach, including consideration of the public interest in a particularly valuable medicinal product).

51 J-S Borghetti, ‘Litigation on Hepatitis B Vaccination and Demyelinating Diseases in France: Braking through Scientific Uncertainty’ in M Martín-Casals and DM Papayannis (eds), *Uncertain Causation in Tort Law* (CUP 2016) 38.

52
*Gee* (n 47).

53 ibid, [152]–[153], [161]. In her view, all the relevant circumstances noted by Hickinbottom J could be taken into consideration in determining defectiveness, including, avoidability, risk–benefit, and cost. ibid, [144]–[167].

54 Consumer Protection Act 1987, s 3(2)(b).

55
*Gee* (n 47) [164].

56 ibid, [164].

57 ibid, [162].

58 cf D Fairgrieve and others, ‘Products in a Pandemic: Liability for Medical Products and the Fight against COVID-19’ (2020) 11 European Journal of Risk Regulation 565, 587 (who equate ‘effectiveness’ with ‘enhanced efficacy’).

59 See B Haynes, ‘Can It Work? Does It Work? Is It Worth It? (1999) 319 British Medical Journal 652; J Marley, ‘Efficacy, Effectiveness, efficiency’ (2000) 23 Australian Prescriber 114.

60 C Zimmer, ‘2 Companies Say Their Vaccines Are 95% Effective. What does That Mean?’ *The New York Times* (New York, 21 November 2020) Section A 10, citing Naor Bar-Zeev, epidemiologist at John Hopkins Bloomberg School of Public Health.

61
*Bailey* (n 47) [14]. See also the adoption of risk–benefit balancing in Scotland by the Inner House of the Court of Session in *Hastings v Finsbury Orthopaedics Ltd* [2021] CSIH 6, 2021 SLT 187 [71] (hip-replacement prosthesis).

62 Fairgrieve and others (n 58).

63
*Bailey* (n 47) [8].

64 ibid, 538.

65 CPA, s 3(2)(c).

66 COVID-19 Genomics UK Consortium, ‘COG-UK Report on SARS-CoV-2 Spike Mutations of Interest in the UK’, 15 January 2021 <https://www.cogconsortium.uk/wp-content/uploads/2021/01/Report-2_COG-UK_SARS-CoV-2-Mutations.pdf> accessed 19 March 2021.

67 Yet not exclusively. Risk–utility would be a central factor in determining defectiveness, but clearly so would the absence of a warning. Risk–utility does not trump a failure to warn of a known risk: cf Fairgrieve and others (n 58) 565, 587, 588.

68 See generally, Fairgrieve and Goldberg (n 42) 10.42–10.45. Indeed, the Product Liability Directive’s drafter, Taschner, was extremely critical of the risk–utility approach as a test for design defectiveness: see H-C Taschner, ‘Product Liability: Basic Problems in a Comparative Law Perspective’ in D Fairgrieve (ed), *Product Liability in Comparative Perspective* (CUP 2005) 160.

69 BGH, 16 June 2009, VI ZR 107/08, 18. See also the importance attached by the Berlin Higher Regional Court to a risk–utility analysis in determining defectiveness (20 U 115/17, 27 May 2019 [23], [28] (metal on metal prosthesis)).

70 Taschner, one of the principal architects of the Product Liability Directive, has conceded that risk–utility may be *relevant* to determining the safety which the public at large legitimately expects: Taschner (n 68) 161.

71 See §84 AMG 1976, § 25(2)5 AMG 1976, and § 4 nos 27, 28 AMG 1976 (discussed in E Rajneri and others, ‘Remedies for Damage Caused by Vaccines: A Comparative Study of Four European Legal Systems’ (2018) 1 European Review of Private Law 57, 70, 71).

72 OLG Hamm, 18 June 2003, 3 U 99/02, NJW-RR 2003, 1382, cited in Rajneri and others, ibid 57, 74.

73 This risk–benefit approach had been adopted in two previous decisions involving medicinal products, viz Isomeride (Versailles, 17 March 2006, no 04/08435; Paris, 19 June 2009, no 06/13741) and in three cases involving the vaccine against hepatitis B (Versailles, 16 March 2007, no 05/09525; 29 March 2007, no 06/00496; 5 November 2007, no 06/106435); see also the recent adoption of risk–benefit by the Cour de cassation in a case concerning the oral contraceptive Adapal: Cass civ 1, 26 September 2018, no 17-21.271 (discussed at n 190).

74 Cass civ 1re, 26 September 2012, no 11-17.738. See also Cass civ 1re, 9 July 2009, no 08-11073, Bull civ I, no 176, D 2010, 49, obs P Brun, JCP G 2009, 308, note P Sargos, RTD civ 2009, 735, obs P Jourdain, RDC 2010, 79, obs J-S Borghetti.

75 Cour d’appel de Versailles, 10 February 2011.

76 Cass civ 1re, 26 September 2012, no 11-17.738.

77 J-S Borghetti, ‘Qu’est-ce qu’un vaccin défectueux?’, Recueil Dalloz 2012, 2853. This case-by-case approach of examining all elements at hand when considering the product’s defectiveness and the existence of a causal link was upheld by the Cour de cassation: Cass civ 1re, 10 July 2013, no 12-21314. For criticism of this approach as being ‘neither convincing nor satisfying’, see J-S Borghetti, ‘Causation in Hepatitis B Vaccination Litigation in France: Breaking through Scientific Uncertainty’ (2016) 91 Chicago-Kent Law Review 543, 557, n 58.

78 Cass civ 1re, 12 November 2015, no 14-18118.

79 Case C-621/15 *NW and others v Sanofi Pasteur MSD SNC*, ECLI:EU:C:2017:484 (21 June 2017).

80 See J Meltzer and C Derycke, ‘Latest CJEU Decision under the Product Liability Directive: National Courts Given a Wide Discretion in Deciding What Claimants Have To Do To Prove Defect and Causation’ (2017) 67 International Product Liability Review 1; LR Smillie and others, ‘C-621/15-*W And Others v Sanofi Pasteur*: An Example of Judicial Distortion and Indifference to Science’ (Commentary) (2018) 26 Medical Law Review 134.

81 Case C-621/15 (n 79) [43].

82 Viz, ‘temporal proximity between the administration of a vaccine and the occurrence of a disease and the lack of personal and familial history of that disease, together with the existence of a significant number of reported cases of the disease occurring following such vaccines being administered’: ibid, [41].

83 Meltzer and Derycke (n 80) 1, 6. While establishment of a true excess risk of an adverse effect could be relevant to a determination of both *causation* (under art 4) and *defectiveness* (under art 6) (through failure to warn of the excess risk) (see *XYZ v Schering Health Care Limited* [2002] EWHC 1420, (2002) 70 BMLR 88 (QB), [21]), the concepts remain distinct.

84 Case C-621/15 (n 79) [36], [37]. In *Gee* (n 47) [80] whilst Mrs Justice Andrews noted the discussion in *NW and others v Sanofi Pasteur MSD SNC* (CJEU) cautioning against applying evidentiary rules in this way, she eschewed the matter of conflation of defect and causation.

85 [2016] EWHC 3096 (QB) [65].

86 ibid, [82].

87 ibid, [83].

88 ibid, [89].

89 ibid, [97].

90 ibid, [101].

91 ibid, [102], [103].

92 ibid, [66].

93 Case C-621/15 (n 79) [85], [86] (opinion of AG Bobek).

94 ibid, [87].

95 ibid, [88].

96 [2018] EWHC 1208 (QB).

97 ibid, [150].

98 ibid, [151].

99 Rajneri and others (n 71) 57, 88.

100 See Directive, art 6 and CPA 1987, s 3(2).

101 Vaccine hesitancy is defined as the ‘delay in acceptance or refusal of vaccines despite availability of vaccine services’: WHO, Report of the SAGE working group on vaccine hesitancy, 1 October 2014 <https://www.who.int/immunization/sage/meetings/2014/october/1_Report_WORKING_GROUP_vaccine_hesitancy_final.pdf> accessed 24 September 2020, 7.

102 IPSOS Survey for The World Economic Forum, 'Global Attitudes on a COVID-19 Vaccine',<https://www.ipsos.com/en/global-attitudes-covid-19-vaccine-december-2020> accessed 15 January 2021; Between 25% in Brazil and 66% in Japan of those who will not take the vaccine mention side-effects: IPSOS Survey for The World Economic Forum, 'Global Attitudes on a COVID-19 Vaccine', <https://www.ipsos.com/en/global-attitudes-covid-19-vaccine-january-2021> accessed 15 January 2021.

103 IPSOS Survey for The World Economic Forum, 'Global Attitudes on a COVID-19 Vaccine', <https://www.ipsos.com/en/global-attitudes-covid-19-vaccine-january-2021> accessed 15 January 2021.

104 cf *Wilkes* (n 39) [2018] QB, [67], [82] (Hickinbottom J).

105 This concerned the Salk killed polio vaccine produced by inactivating poliovirus with formaldehyde, and as a result of inadequate safety testing and filtration methods to inactivate the virus, at least 220,000 people were infected with the virus, 70,000 developed muscle weakness, 164 were severely paralysed, and 10 killed: P Offit, ‘The Cutter Incident, 50 Years Later’ (2005) 352 New England Journal of Medicine 1411; P Offit, *The Cutter Incident* (Yale University Press 2005) 89.

106 Note, ‘The Cutter Polio Vaccine Incident: A Case Study of Manufacturers Liability Without Fault in Tort and Warranty’ (1955) 65 Yale Law Journal 262.

107
*Kearl v Lederle Laboratories* 218 California Reporter 453, 458 (Cal App, 1985).

108 (1955) 65 Yale Law Journal 262, 273 (emphasis in original).

109 E Parasidis, ‘Recalibrating Vaccination Laws’ (2017) 97 Boston University Law Review 2153, 2171; Offit, *The Cutter Incident* (n 105) 133.

110 6 California Reporter 320, 322 (California District Court of Appeal, 1960).

111 The case concerned the Salk killed polio vaccine produced by inactivating poliovirus with formaldehyde, and as a result of inadequate safety testing and filtration methods to inactivate the virus, at least 220,000 people were infected with the virus, 70,000 developed muscle weakness, 164 were severely paralysed, and 10 killed: Offit, The Cutter Incident, 50 Years later (n 105); Offit, *The Cutter Incident* (n 105) 89.

112
*Gottsdanker* (n 110) 320, 322, 325, 326.

113 ibid 322.

114 ibid 326.

115 ibid 325, 326.

116
*Kearl* (n 107)

117 See Restatement (Second) of Torts 402A(1) and (2)(a) (American Law Institute 1965) and, in general, Owen and Davis, *Products Liability*, 19.4.

118 See R Goldberg, *Medicinal Product Liability and Regulation* (Hart Publishing 2013) 39–42.

119 218 California Reporter 453 (Cal App, 1985).

120 ibid 458, 459.

121 ibid 459.

122 See Restatement (Second) of Torts 402A cmt *k* (American Law Institute 1965). For the genesis of Comment *k* and how it came to favour the non-liability of manufacturers for drug design, see JA Henderson and AD Twerski, ‘Drug Design Liability: Farewell to Comment k’ (2015) 67 Baylor Law Review 521, 523–32.

123 498 F 2d 1264 (5th Cir 1974), cert denied, 419 US 1096 (1974).

124 ibid 1274. See further, *Needham v White Labs*, 639 F 2d 394, 401 (7th Cir 1981).

125 See Henderson and Twerski (n 122) 521, 530–32, 542–54.

126
*Kearl* (n 107) 453, 463, 464 (Cal App, 1985).

127 ibid 464.

128 ibid 463.

129 Henderson and Twerski (n 122) 521, 542–49.

130 ibid 546; cf *Brochu v Ortho Pharmaceutical Corp* (1981) 642 F 2d 652, 655 (oral contraceptive with 100 mg oestrogen; Ortho marketing four other contraceptives equally effective but presenting much less risk with lower oestrogen content); *Frazier v Mylan, Inc*, 911 F Supp 2d 1285 (ND Ga 2012) (phenytoin; four well-known safer alternative FDA-approved seizure drugs with better safety profile and lower risk; allegations of substitute products for phenytoin could be sufficient under a risk–utility analysis).

131 732 P 2d 297, 311 (Idaho 1987).

132 ibid 300–01.

133 779 F2d 1429, 1433 (9th Cir 1986).

134 732 P 2d 297, 310–11.

135 ibid 310.

136 ibid 311.

137 732 P 2d 297, 301; 779 F2d 1429, 1430–31.

138 Henderson and Twerski (n 122) 521, 548.

139 ibid.

140
*Institute of Medicine: Vaccine Supply and Innovation: Report of the Committee on Public-Private Sector Relations in Vaccine Innovation* (National Academy Press 1985), 115.

141 ibid.

142
*Bruesewitz v Wyeth LLC* (2011) 562 US 223, 227.

143 ibid.

144 42 USC 300aa-1; See HR Rep No 99-908, 4–7 (1986); and, further, JB Apolinsky and JA Van Detta, ‘Rethinking Liability for Vaccine Injury’ (2010) 19 Cornell Journal of Law and Public Policy 537, 550–51. The ‘great difficulty’ in manufacturers obtaining affordable product liability insurance to cover losses relating to vaccine injuries was the stated reason for one manufacturer withdrawing from the vaccine market in 1984: HR Rep No 99-908, 6 (1986).

145 HR Rep No 99-908, 26 (1986) (‘The Committee has set forth Comment *K* in this bill because it intends that the principle in Comment *K* regarding “unavoidably unsafe” products, i.e, those products which in the present state of human skill and knowledge cannot be made safe, apply to the vaccines covered in the bill and that such products not be the subject of liability in the tort system’).

146 42 USC 300aa-22(b)(1).

147 562 US 223, 256 (2011) (dissenting Opinion of Sotomayor J (joined by Ginsburg J)).

148 562 US 223, 232–37 (Opinion of the Court (Scalia J)). The subsequent argument was that having established that the preemption clause invoked Comment *k*, both Comment *k* and the Vaccine Act did not prevent the petitioners from establishing liability for defective design by proving that a reasonable (and safer) alternative design to Wyeth’s whole-cell DPT vaccine was available: ibid 258, 261 (Sotomayor J (dissenting, joined by Ginsburg J)) and Brief for Kenneth W Starr and others as *Amici Curiae* 14–15. cf Opinion of the Court (Scalia J), 241; ibid 245 (Breyer J, concurring).

149 562 US 223, 231–32 (2011).

150 42 USC 300aa-22(b)(1).

151
*Bruesewitz* (n 142) 223, 243. For criticism of the Supreme Court’s decision as the finding of preemption in a ‘hyper-textual analysis’, assessing Congressional intent solely through the text of express preemption , see MJ Davis, ‘The Case Against Preemption: Vaccines and Uncertainty’ (2011) 8 Indiana Health Law Review 293, 307, 313. While Justice Scalia did not describe 300aa-22(b)(1) as an express preemption clause, it can be inferred that he considered that the clause ‘provided sufficient evidence of a clear and manifest preemptive purpose to qualify it as express preemption’: MS Holland, ‘Liability for Vaccine Injury: The United States, the European Union, and the Developing World’ (2018) 67 Emory Law Journal 415, 439.

152 Note that the NCVIA only applies to vaccines listed on the Vaccine Injury Table (42 USC 300aa-11(b)(1)(A)) and is inapplicable to several vaccines, such as the shingles vaccine for adults. Those individuals injured by these vaccines can continue to bring civil claims.

153
*Bruesewitz* (n 142) 223, 250 (Sotomayor J, dissenting).

154 HR Rep No 99-908 (1986) 26.

155
*Bruesewitz* (n 142) 223, 268, 269 (Sotomayor J, dissenting); cf (n 142) 223, 237, 238 (majority opinion).

156 ibid 238 (majority opinion). Those means to develop improved designs are: the responsibility of the Secretary of HHS ‘to promote the development of childhood vaccines that result in fewer and less serious adverse reactions’ (42 USC 300aa-27(a)(1)), the establishment of a National Vaccine Program ( 300aa-1-3), and the requirement of healthcare providers and vaccine manufacturers to report adverse effects (( 300aa-25(b)): 562 US 223, 238, 239.

157 ibid 269, 270 (Sotomayor J, dissenting). See also Holland (n 151) 415, 440–43.

158 ibid 269, 270 (Sotomayor J, dissenting).

159 ibid 270 (Sotomayor J, dissenting) (citing *Wyeth v Levine* (2009) 555 US 555, 578, 579). Without the ‘safety net’ of civil litigation, there are arguments to support the amendment and strengthening of the NCVIA with ‘additional regulatory support’: A Kesselheim, ‘Safety, Supply and Suits-Litigation and the Vaccine Industry’ (2011) 364 New England Journal of Medicine 1485, 1487; and, further, A Kesselheim, ‘The Role of Litigation in Defining Drug Risks’ (2007) 297 New England Journal of Medicine 308, 311 (limiting prescription drugs litigation likely to increase ‘poorly defined or inadequately presented drug risk information’).

160 See Davis (n 151) 293, 296, 315, 316. *Bruesewitz* has heightened the feeling of families and vaccine critics that they are forced to pursue their claims in a hostile forum, instead of being able to sue in court with the right to discovery in front of a jury: A Kirkland, *Vaccine Court: The Law and Politics of Injury* (New York University Press 2016) 195.

161
*Bruesewitz* (n 142) 223, 238.

162 ibid 223, 229.

163 Kesselheim (n 159)‘Safety, Supply and Suits’.

164 See the position where parents were able to bypass the NCVIA and sue the manufacturers of Thimerosal, a preservative added to vaccines by the vaccine manufacturers, in the Federal Court: *Holder v Abbott Laboratories*, Inc 444 F3d 383, 389 (5th Cir 2006). For criticism of this gap as a weakness of the NCVIA, see Offit, *The Cutter Incident* (n 105)184–86, and, further, PM Danzon and NS Pereira, ‘Vaccine Supply: Effects of Regulation and Competition’ (2011) 18 International Journal of the Economics of Business 239, 245, 246 (noting that the legal costs of defending against such claims and the risk they might eventually succeed may act as a disincentive to vaccine entry).

165 See further, M Herrmann and others, *Drug and Device Product Liability Litigation Strategy* (2nd edn, OUP 2018) 11.111–11.112.

166 That is, declaring a drug to be defectively designed if, from an overall perspective, its risk outweighs its benefits: Henderson and Twerski (n 122) 521, 549–51 (macro-risk-utility would require a complex evidentiary inquiry of the overall social value of a drug; and if a drug’s overall risks outweigh its overall benefits, then even if the drug was highly valuable for one or more distinct classes of users, the court might still strike down the design as defective and not worthy of being prescribed, including for those who would benefit from it).

167 That is, determining only whether a drug is reasonably safe for the *particular class* of patients for which the plaintiff is a member, would signal that the drug should be changed for them but not for others: this would result in the drug being continued to be marketed with strengthened warnings or the company would withdraw the drug from the market, with the consequent deprivation of potentially a majority of its users: ibid 551–53.

168 The alternative Restatement (Third) of Torts: Products Liability 6(c) supported by Co-reporters Henderson and Twerski, adopting a net-benefit test provides that a drug is defectively designed if no reasonable healthcare provider, knowing of the drug’s foreseeable risks and benefits, would prescribe the drug for any class of patients. This ‘individualized, nonaggregative approach to benefit–risk analysis’ permits a minority class of patients to have access to the drug: if the drug provides a positive benefit–risk ratio such that a reasonable healthcare provider would prescribe it for one class of patients, it is not defective in design: ibid 532–35.

169 Though that would be difficult to establish. In comparing the Sabin OPV (using an attenuated form of the viral agent) with the Salk IPV (using a killed virus), while OPV carried a one-in-2.4 million risk of causing vaccine-associated paralytic polio, there were in fact intestinal immunity advantages with OPV and only OPV can prevent infection: L Noah, ‘This is Your Products Liability Restatement on Drugs’ (2009) 74 Brooklyn Law Review 839, 876–78.

170 See Amicus Brief of Professor Mark Geistfeld, *Bruesewitz v Wyeth LLC*, 1 June 2010, 4.

171 cf the UK Vaccine Damage Payments Act 1979, s 6(4) (no fault compensation scheme without prejudice to the ability to pursue civil proceedings against the manufacturer of the vaccine).

172 Karafillakis and Larson (n 31).

173 Department of Health & Social Care, *Consultation Document: Changes to Human Medicines Regulations to Support the Rollout of COVID-19 Vaccines* (28 August 2020) 3, 6, 8.

174 See Human Medicines Regulations 2012, SI 2012/1916, reg 174, as amended by the Human Medicines (Coronavirus and Influenza) (Amendment) Regulations 2020, SI 2020/1125, reg 6. The basis of this lies in Dir 20001/83/EC (the Medicinal Products Directive) [2001] OJ L311/67, as amended by Dir 2004/27/EC [2004] OJ L136/34, art 5(2).

175 MHRA Press release: 'UK medicines regulator gives approval for first COVID-19 vaccine', <https://www.gov.uk/government/news/uk-medicines-regulator-gives-approval-for-first-uk-covid-19-vaccine> accessed 3 December 2020.

176 MHRA Press release: 'Oxford University/Astra Zeneca COVID-19 vaccine approved', <https://www.gov.uk/government/news/oxford-universityastrazeneca-covid-19-vaccine-approved> accessed 30 December 2020.

177 MHRA Press release: 'Moderna vaccine becomes third COVID-19 vaccine approved by UK regulator', <https://www.gov.uk/government/news/moderna-vaccine-becomes-third-covid-19-vaccine-approved-by-uk-regulator> accessed 8 January 2021.

178 Dir 20001/83/EC (the Medicinal Products Directive) [2001] OJ L311/67, as amended by Dir 2004/27/EC [2004] OJ L136/34, art 5(3).

179 See Human Medicines Regulations 2012, SI 2012/1916, reg 345(3), as amended by the Human Medicines (Coronavirus and Influenza) (Amendment) Regulations 2020, SI 2020/1125, reg 29.

180 See Human Medicines Regulations 2012, SI 2012/1916, reg 345(4).

181 See D Fairgrieve and others, ‘Response to DHSC Open Consultation: Changes to Human Medicines Regulations to Support the Rollout of COVID-19 Vaccines’ (18 September 2020) paras 12, 13. Controversially, art 14.1 of the published Advance Purchase Agreement between the European Commission and AstraZeneca reveals a wide indemnity clause, obliging each participating Member State to indemnify AstraZeneca from ‘any and all damages and liabilities, including settlements’, and necessary legal costs. The indemnity clause in the Supply Agreement between AstraZeneca UK and the Secretary of State for Business, Energy and Industrial Strategy of 28 August 2020 is entirely redacted.

182 In *Bailey* (n 47) [43], the Court of Appeal upheld the need for ‘absolute clarity’ in defining the alleged defect, since it is the defect that must cause the injury, and it is in respect of the defect that the defendant can raise the development risk defence: the case on defect also drives the scope of the expert evidence.

183
*Wilkes* (n 39), [2018] QB, [67], [78], [82].

184 See the discussion on the use of a vaccine to achieve herd immunity safely from the SARS-Cov-2 virus by the WHO’s Chief Scientist, Dr Soumya Swaminathan: <https://www.who.int/emergencies/diseases/novel-coronavirus-2019/media-resources/science-in-5/episode-1> accessed 29 September 2020.

185
*Bailey* (n 47) [48].

186 ibid.

187
*Bailey* (n 47) [14].

188 ibid, [27], [48].

189 Thus, following the roll-out of the the COVID-19 Vaccine AstraZeneca, large-scale monitoring revealed a possible link between the vaccine and extremely rare and unlikely to occur blood clots, and suggested a slightly higher incidence in younger adult age groups, but the MHRA has concluded that the benefits of vaccination continue to outweigh any risks. In communicating the potential benefits and harms of the AstraZeneca vaccine, benefits noted include protection against COVID-19 (short-term and ‘long COVID’) for the person vaccinated and those they come into contact with <https://www.gov.uk/government/news/mhra-issues-new-advice-concluding-a-possible-link-between-covid-19-vaccine-astrazeneca-and-extremely-rare-unlikely-to-occur-blood-clots> accessed 7 April 2021; <https://wintoncentre.maths.cam.ac.uk/news/> accessed 7 April 2021.

190 See the decision of the French Cour de cassation concerning the death of a 25-year-old female from a massive pulmonary embolism, allegedly caused by the oral contraceptive Adapal. The court took into consideration the benefit–risk ratio but held that, despite the benefits conferred by the contraceptive pill, and the presence of a warning of the risk of pulmonary embolism, the ability to cause death in an otherwise young healthy woman shifted the scales in favour of finding the product defective: Cass civ 1, 26 September 2018, no17-21.271. For the difficulty in reconciling this 2018 decision of the Cour with the previous 2012 and 2013 decisions concerning the hepatitis B vaccine litigation, see MS Silva and others, ‘Relevance of Risk-Benefit for Assessing Defectiveness of a Product: A Comparative Study of Thirteen European Legal Systems’ (2021) 29 European Review of Private Law 91, 105, 106.

191
*Wilkes* (n 39) [82], [83]; approved in *Gee* (n 47) [167].

192 See n 169.

193 H Ledford, ‘Why COVID Vaccines Are So Difficult to Compare’ (2021) 591 Nature 16, 17.

194 DeStefano and others (n 22).

195
*Wilkes* (n 39) [66] (emphasis added).

196 See text to n 31.

197 Karafillakis and Larson (n 31).

